# Chronic Obstructive Pulmonary Disease heterogeneity: challenges for health risk assessment, stratification and management

**DOI:** 10.1186/1479-5876-12-S2-S3

**Published:** 2014-11-28

**Authors:** Josep Roca, Claudia Vargas, Isaac Cano, Vitaly Selivanov, Esther Barreiro, Dieter Maier, Francesco Falciani, Peter Wagner, Marta Cascante, Judith Garcia-Aymerich, Susana Kalko, Igor Marin De Mas, Jesper Tegnér, Joan Escarrabill, Alvar Agustí, David Gomez-Cabrero, the Synergy-COPD consortium

**Affiliations:** 1 IDIBAPS, Hospital Clínic. Facultat de Medicina, 08036, Barcelona, Catalunya, Spain; 2 Centro de Investigación Biomédica en Red de Enfermedades Respiratorias (CIBERES), Bunyola, Balearic Islands; 3 Pulmonology Department-Muscle and Respiratory System Research Unit (URMAR), IMIM-Hospital del Mar, Health and Experimental Sciences Department (CEXS), Universitat Pompeu Fabra (UPF), Parc de Recerca Biomédica de Barcelona (PRBB), Barcelona, Catalonia, Spain; 4 Biomax Informatics AG, 282152 Planegg, Germany; 5 Centre for Computational Biology and Modeling (CCBM). Institute of Integrative Biology. University of Liverpool.Crown Street.L69 7ZB.UK; 6 Division of Physiology, Pulmonary and Critical Care Medicine, University of California, San Diego, La Jolla, CA, USA; 7 Departament de Bioquimica i Biologia Molecular i IBUB, Facultat de Biologia, Universitat de Barcelona, 08028 Barcelona, Spain; 8 Centre for Research in Environmental Epidemiology (CREAL). CIBER Epidemiología y Salud Pública (CIBERESP). Universitat Pompeu Fabra, Departament de Ciències Experimentals i de la Salut Barcelona, Spain; 9 Unit of Computational Medicine, Department of Medicine, Center for Molecular Medicine, Karolinska Institutet, Karolinska University Hospital, Stockholm, Sweden

**Keywords:** Chronic diseases, COPD, Disease heterogeneity, Integrated Care, Predictive Medicine, Redox disequilibrium, Systems Medicine, VO_2_max

## Abstract

**Background and hypothesis:**

Heterogeneity in clinical manifestations and disease progression in Chronic Obstructive Pulmonary Disease (COPD) lead to consequences for patient health risk assessment, stratification and management. Implicit with the classical *"spill over" *hypothesis is that COPD heterogeneity is driven by the pulmonary events of the disease. Alternatively, we hypothesized that COPD heterogeneities result from the interplay of mechanisms governing three conceptually different phenomena: 1) pulmonary disease, 2) systemic effects of COPD and 3) co-morbidity clustering, each of them with their own dynamics.

**Objective and method:**

To explore the potential of a systems analysis of COPD heterogeneity focused on skeletal muscle dysfunction and on co-morbidity clustering aiming at generating predictive modeling with impact on patient management. To this end, strategies combining deterministic modeling and network medicine analyses of the Biobridge dataset were used to investigate the mechanisms of skeletal muscle dysfunction. An independent data driven analysis of co-morbidity clustering examining associated genes and pathways was performed using a large dataset (ICD9-CM data from Medicare, 13 million people). Finally, a targeted network analysis using the outcomes of the two approaches (skeletal muscle dysfunction and co-morbidity clustering) explored shared pathways between these phenomena.

**Results:**

(1) Evidence of abnormal regulation of skeletal muscle bioenergetics and skeletal muscle remodeling showing a significant association with nitroso-redox disequilibrium was observed in COPD; (2) COPD patients presented higher risk for co-morbidity clustering than non-COPD patients increasing with ageing; and, (3) the on-going targeted network analyses suggests shared pathways between skeletal muscle dysfunction and co-morbidity clustering.

**Conclusions:**

The results indicate the high potential of a systems approach to address COPD heterogeneity. Significant knowledge gaps were identified that are relevant to shape strategies aiming at fostering 4P Medicine for patients with COPD.

## Introduction

### COPD is a highly heterogeneous major chronic disease

Chronic Obstructive Pulmonary Disease (COPD) is a prevalent chronic disorder caused by the inhalation of irritants, mainly tobacco smoke. It affects approximately 9% of the adult population above 45 yrs [1]. The disease imposes a high burden on healthcare systems worldwide and it is currently the fourth leading cause of mortality [2]. In 2001, the World Health Organization (WHO) included COPD among the five major chronic disorders together with cardiovascular diseases, cancer, diabetes and mental disorders [3].

Current diagnosis of COPD is based on three concurrent criteria [4]: *i) *presence of respiratory symptoms, mainly shortness of breath and/or chronic cough/sputum; *ii) *history of inhalation of irritants; and, *iii) *forced spirometry testing indicating an obstructive ventilatory defect. The expiratory flow limitation observed in COPD patients is due to increased airways resistance and/or reduced lung elasticity caused by destruction of pulmonary parenchyma.

Approximately 15 to 20% of all tobacco smokers are prone to develop COPD and there is marked individual variability of both clinical manifestations and pulmonary disease progression [5-7] with relevant implications in terms of health risk assessment and patient management [6,8]. It is well established that COPD patients can present acute episodes of exacerbation with a negative impact on use of healthcare resources and prognosis [6,9]. Moreover, these patients can also show systemic effects of the disease - being skeletal muscle dysfunction/wasting [10] a characteristic one - and co-morbid conditions [6,11]. Highly prevalent chronic conditions such as cardiovascular disorders (CVD) and type 2 diabetes mellitus - metabolic syndrome (T2DM-MS) are often present as a co-morbidity cluster in COPD patients [12-14]. There is evidence that both skeletal muscle dysfunction/wasting and co-morbidity clustering are independently associated with poor prognosis [1,6,10].

### State of the art on COPD heterogeneity and main challenges

The Global initiative for Obstructive Lung Disease (GOLD) [1] has played a major role in raising COPD awareness and defining standards for treatment. GOLD has faced the challenge of COPD heterogeneity by evolving from an initial disease staging based only on the degree of airflow limitation (FEV_1_, forced expiratory volume during the first second) [15] to the incorporation of symptoms and frequency of severe exacerbations into the scoring system (2011 GOLD update) (see Table 1 for details) and acknowledging the negative impact of co-morbid conditions on prognosis. Evidence-based data using the 2011 GOLD classification are currently emerging, but the results are not yet consolidated [16,17]. Alternative available options for COPD classification or prediction of survival [18-22] are also insufficient for *subject-specific *prediction and stratification of management.

**Table 1:** Risk classification of COPD patients according to the 2011 GOLD Update [1]

RISK *GOLD Classification*	3-4	C **High Risk**, Less Symptoms	D **High Risk**, More Symptoms	≥2	RISK *Exacerbation History*
		
	1-2	A **Low Risk**, Less Symptoms	B **Low Risk**, More Symptoms	0-1	
**mMRC 0-1**			**mMRC ≥ 2**		
**CAT < 10**			**CAT ≥ 10**		

The **2011 COPD Update **[1] defines four risk categories for COPD patients (A to D) depending upon: *i) ***symptoms **(modified dyspnea score from th Medical Research Council, mMRC) or CAT questionnaire; *ii) ***spirometric classification**: GOLD I: FEV_1 _≥ 80% pred; GOLD II: 50% ≤ FEV_1 _< 80% pred; GOLD III: 30% ≤ FEV_1 _< 50% pred; and, GOLD IV: FEV_1 _< 30% and/or PaO_2 _< 60 mmHg breathing F_I_O_2 _0.21); and, *iii) ***frequency of exacerbations per year**. Recent reports have assessed the predictive value of this classification [16,17]

ECLIPSE - The Evaluation of COPD Longitudinally to Identify Predictive Surrogate End-points study - has been a 3-year follow-up project of a large cohort of well characterized COPD GOLD II-IV patients [23] that has generated a relevant body of knowledge on several major aspects of the disease. A recent summary report (2014) on clinical implications of the project outcomes [6] stresses the impact of COPD heterogeneity observed in both the cross-sectional and the longitudinal study assessments.

All in all, there is a strong rationale for further research on subject-specific health risk prediction and stratification aiming at enhancing cost-effective management of COPD patients. However, there are several major challenges to be taken into account in the design of a systems approach to better understand COPD heterogeneity. Firstly, the overlap among different chronic obstructive airways diseases (COPD, asthma, bronchiectasis, bronchiolitis, etc...) requires novel disease taxonomies based on a better knowledge of underlying mechanisms that may likely result in a re-definition of COPD as a pulmonary disease [24]. Such an approach should solve three current deficits, namely: *i) *overlap between COPD and lung ageing; *ii) *availability of operational diagnostic criteria differentiating COPD from other obstructive pulmonary diseases; and, *iii) *a proper capture of pulmonary heterogeneity of the disease. Another major challenge is to clarify the current confusion between systemic effects of COPD and some co-morbid conditions due to the descriptive nature of the reporting. As an example, is anxiety-depression a systemic effect ?, a COPD complication? or a co-morbid condition?. Likely, a proper understanding of the mechanisms involved in the relationships between systemic inflammation in COPD and depression [25,26] may help to clarify this type of questions. To prevent further confusion, Synergy-COPD was focused on the analysis of skeletal muscle dysfunction/wasting as a well accepted paradigm of a systemic effect of COPD. Last, but not least, a major challenge is a proper understanding of the phenomenon of co-morbidity clustering. There is evidence indicating that co-morbidity clustering is only partly explained by shared risk factors among the concurrent diseases [12,27,28], namely: tobacco smoking, unhealthy diet and sedentarism.

### The Synergy-COPD project

As stated in [29] of the current Supplement, the Synergy-COPD project was conceived as a systems approach to explore the role of combined modeling strategies to better understand COPD heterogeneity. Within the core aim of the project was the purpose of transferring the acquired knowledge into healthcare by designing innovative strategies to effectively build-up 4P Medicine: predictive, preventive, personalized and participatory medicine for chronic patients. In this regard, COPD was identified as a proper use case to explore the potential for generalization of the approach to other chronic conditions.

**Figure 1:**
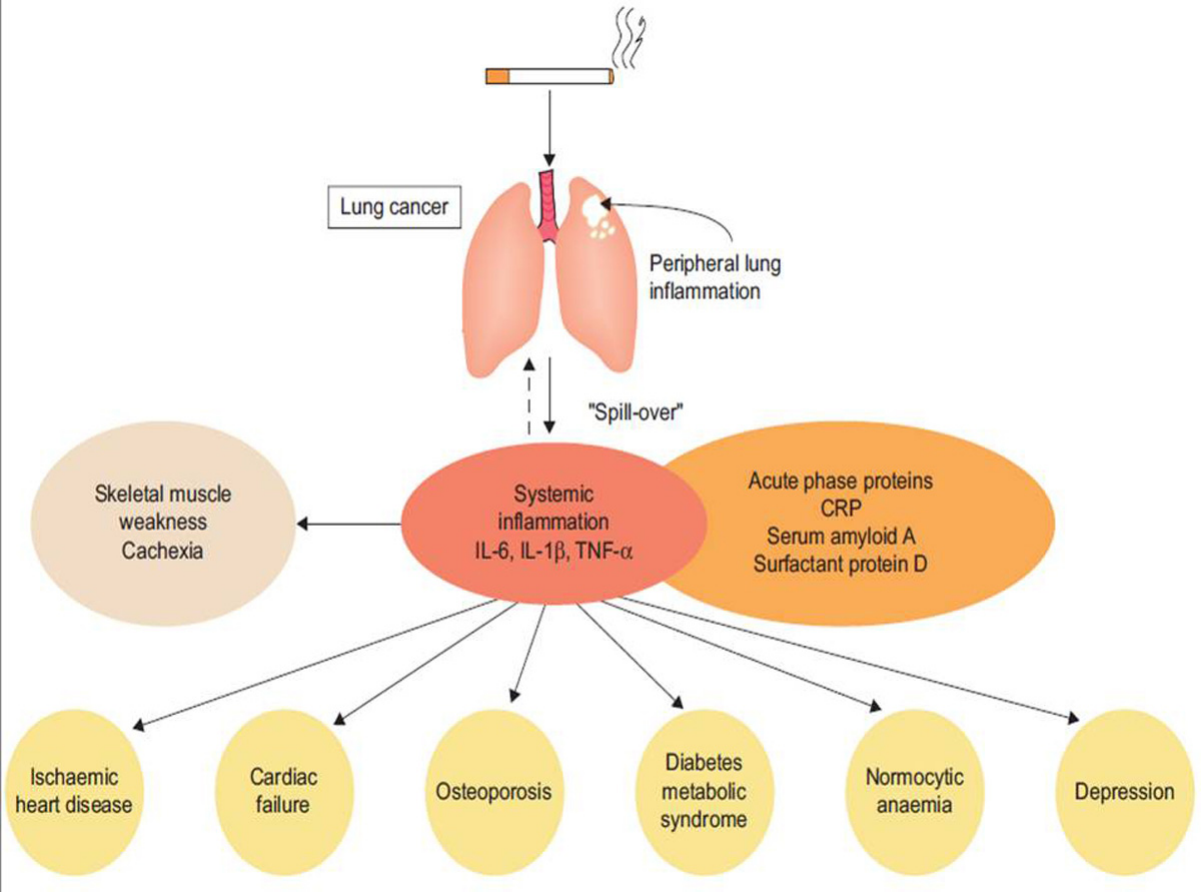
**Lung injury caused by inhaled irritants like tobacco smoking generates peripheral lung inflammation that may cause "spill over" of different types of cytokines into the systemic circulation**. According to this hypothesis, systemic inflammation causes skeletal muscle dysfunction and muscle wasting, but it may also cause and worsen co-morbidities *(reproduced from *[30]*with permission)*

The current chapter focuses on the biomedical dimensions of the project taking into consideration the implications on the healthcare scenario. Figure 1 depicts relevant bio-pathological processes involved in COPD displayed according to the classical *"spill over" *hypothesis [30] to explain the systemic effects of the disease. Two main limitations of this hypothesis are; *i) *its over-simplistic explanation of the phenomenon of systemic low-grade inflammation, not confirmed by ECLIPSE [6,11] and other studies [28] and, *ii) *lack of a proper consideration of the co-morbidity challenge. An implicit assumption of the hypothesis is that COPD heterogeneity is ultimately driven by the pulmonary events of the disease.

Alternatively, Synergy-COPD proposed that COPD heterogeneity is explained by the interplay of conceptually independent events occurring at three different levels: *i) Pulmonary disease *- determined by the effects of lung injury and local remodeling processes; *ii) ***Systemic effects of the disease** with different manifestations, such as skeletal muscle dysfunction/wasting [10]; and, *iii) ***Co-morbidity clustering** that refers to observed associations of different chronic disorders. Synergy-COPD targeted the analysis of associations among CVD, T2DM-MS and COPD. The project explored underlying mechanisms of COPD heterogeneity focusing on skeletal muscle dysfunction/wasting and co-morbidity clustering and it only marginally addressed pulmonary events of the disease.

## Method

### Planned strategies for assessing COPD heterogeneities

The overall biomedical strategy of the project and the specific input datasets have been reported in detail in [29]. Moreover, the different modeling tools and strategies are described in [31]. The three biomedical areas addressed in Synergy-COPD had specific study designs that are summarized below:

***Skeletal muscle dysfunction ***- The project explored three relevant aspects of skeletal muscle dysfunction and muscle wasting in COPD patients. Firstly, Synergy-COPD examined the degree of association between classical COPD GOLD stages (I to IV) and estimations of both cellular oxygenation (PmO_2_) and mitochondrial reactive oxygen species (ROS) levels in skeletal muscle exercising maximally (VO_2_max). The study was done using a COPD dataset wherein VO_2_max, cardiac output (Q_T_), pulmonary ventilation-perfusion mismatching (V_A_/Q inequalities) and blood oxygenation, arterial and mixed venous blood, had been measured [32]. The analysis was carried out using the integrated deterministic model developed in the project wherein an integrated physiological O_2 _pathway model was made interoperable with a biochemical model characterizing mitochondrial ROS generation, as reported in detail in [31]. Likewise, such an analysis was also done using the Biobridge dataset [33] wherein healthy subjects and COPD patients had a multilevel (omics, biochemical, physiological and clinical data) characterization of lower limb muscle, blood and whole-body changes from pre- to post- high intensity supervised resistance training during 8 weeks.

A second study in the project enriched the initial network medicine analysis [34-36]) from the Biobridge dataset [33] by including additional "omics" information [27,28], as well as an extended set of measurements on nitroso-redox balance carried out in blood and in skeletal muscle [28]. The purpose of the network approach described in detail in [31] was to compare healthy subjects and COPD patients in terms of the relationships among relevant metabolic pathways governing cellular bioenergetics, protein balance, and skeletal muscle remodeling paying particular attention to the role of nitroso-redox disequilibrium in the network modeling.

Finally, a third study undertaken within Synergy-COPD addressed the analysis of abnormal training adaptations comparing healthy subjects and COPD from the Biobridge database. Two different modeling strategies were undertaken: targeted probabilistic network modeling [34,37,38] and a Thomas network approach, as described in detail in [31]. In the former, associations between estimated skeletal muscle ROS levels obtained with the integrated deterministic model were compared with actual measurements pre- and post-training carried out both in blood and skeletal muscle.

**Co-morbidity analyses **- Two different studies were undertaken. Firstly, a data driven approach aimed at assessing different indices of relative risk for co-morbidity clustering in COPD patients aged +65 yrs compared to non-COPD patients. As reported in [29], the study was done using the Medicare dataset (13 million people) [29,31]. The research also identified genes and pathways associated with clusters of co-morbidities. A second independent study compared the outcomes of the data driven study with the pathway analysis of the co-morbidity clustering targeted in Synergy-COPD, namely: CVD, T2DM-MS and COPD as reported in [12,13]. The relevant pathways identified in the analysis of skeletal muscle dysfunction/wasting were compared with those seen in the co-morbidity clustering to explore commonalities.

**Pulmonary events **- In 2011, the PAC_COPD study [39] reported an unbiased cluster analysis identifying subtypes of COPD patients with clinical and prognostic implications. In the study [39], there was evidence of a dissociation between relatively low central airways resistance and high emphysema score in approximately one third of the patients. Because of the potential interest of the finding in terms of patient stratification, we used modeling techniques exploring spatial pulmonary heterogeneities to address the issue, as described in detail in [40]. The study was done in close collaboration with the AIRPROM project.

### Health risk assessment and patient stratification

The design of strategies aiming at speeding the transfer of biomedical research achievements into clinical practice constitutes a core objective of Synergy-COPD. To this end, three specific goals were identified: *i) *to better understand the underlying biological mechanisms of the phenomena indicated above, namely: skeletal muscle dysfunction, co-morbidity clustering and dissociation between airway remodeling and emphysema score in COPD patients; *ii) *to identify combined markers with potential predictive power; and, *iii) *to construct patient-specific predictive modeling useful for the clinical decision making processes in primary care. The project outcomes from these three areas should help to generate rules feeding Clinical Decision Support Systems (CDSS) embedded into the clinical processes in primary care. The CDSS produced in the project targeted the areas displayed in Table 2. They were embedded into clinical processes supported by the Integrated Care Shared Knowledge Platform [41] deployed in the integrated healthcare district of Barcelona-Esquerra. Specific validation strategies were defined and executed, as reported in detail in [42].

**Table 2:** Clinical Decision Support Systems (CDSS) developed in Synergy-COPD for COPD management in an integrated care scenario.

**1. Early diagnosis - COPD case finding program **The suite of CDSS supports the regional deployment of a program of early COPD diagnosis targeting citizens at risk examined in pharmacy offices and non-diagnosed patients studied in primary care. Additional objectives of the program are to ensure high quality forced spirometry accessible across heathcare tiers, as well as prevention of over-diagnosis of COPD in elderly due to the GOLD diagnostic criteria [1].
**2. Enhanced stratification of COPD patients **It includes three families of CDSS with well differentiated objectives: *i) *enhance applicability of the 2011 GOLD Update criteria for COPD staging [1]; *ii) *facilitate off-line comparisons with other COPD staging criteria, namely: BODE, DOSE, ADO, etc...[18-21]; and, *iii) *enhanced stratification combining acquired knowledge in Synergy-COPD and consolidated findings from ECLIPSE [6].
**3. Community-based integrated care program **The suite of CDSS aims at supporting different integrated care services fostering the transfer of complexity from specialized care to the community with an active role of patients. The two programs being deployed are: *i) *sustainability of training-induced effects and promotion of physical activity; and, *ii) *management of patients under long-term oxygen therapy (LTOT). The two programs were assessed within NEXES [41,49], as part of the deployment of integrated care services in the health district of Hospital Clinic.

## Results

### Contributions to knowledge on COPD heterogeneities

**Figure 2:**
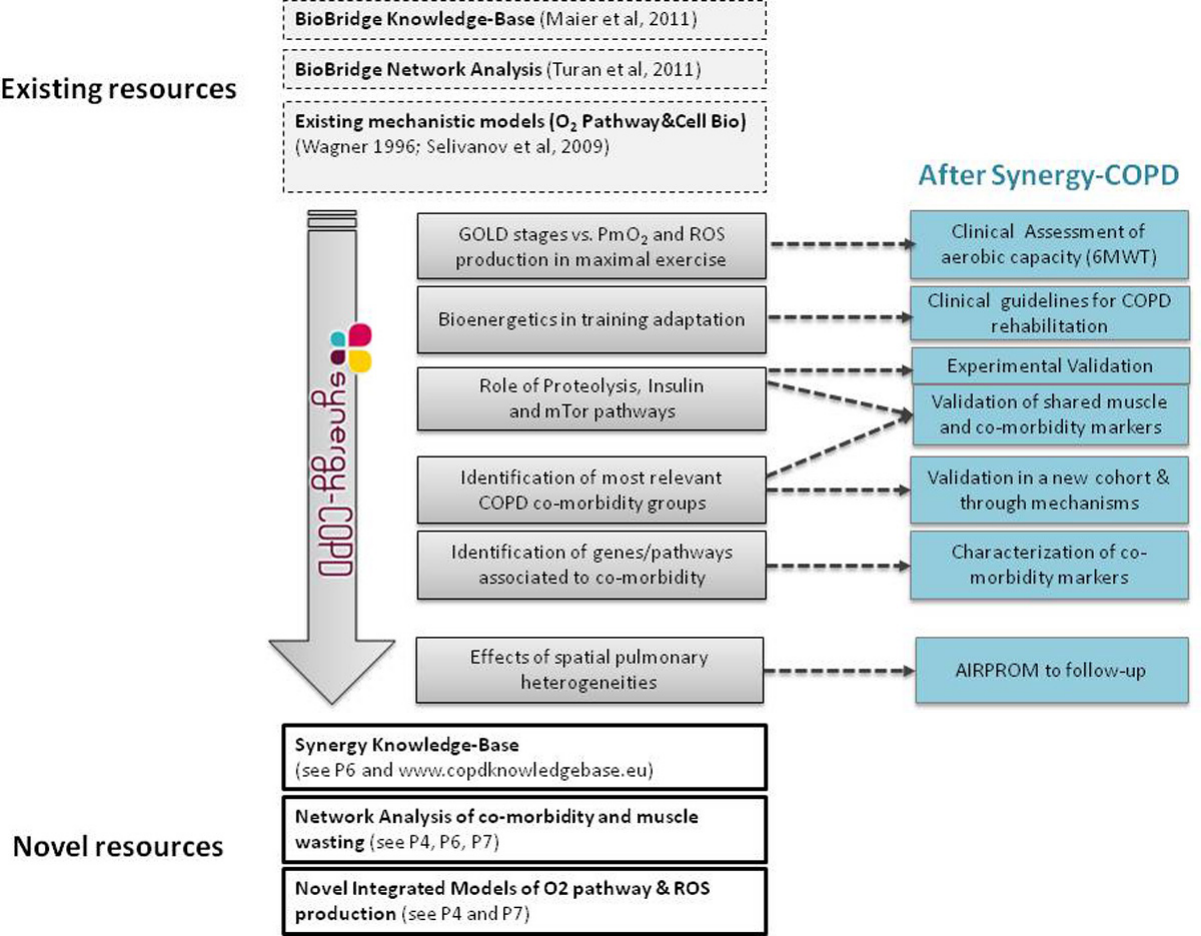
**Diagram indicating input data *(top rectangles with discontinuous line)*, biomedical achievements *(central grey rectangles)*, resources generated by the project *(bottom rectangles with continuous line) *and further developments to be considered after the Synergy-COPD project**. *(blue rectangles)*.

Figure 2 summarizes four major aspects of the Synergy-COPD project: *(i) *main input data for the analyses, also described in detail in [29] and [43]; *(ii) *main biomedical analyses carried out during the project lifetime; *(iii) *novel resources generated from the developments done; and, finally, *(iv) *areas of impact from the project and recommendations to be done beyond the project life span.

**Figure 3:**
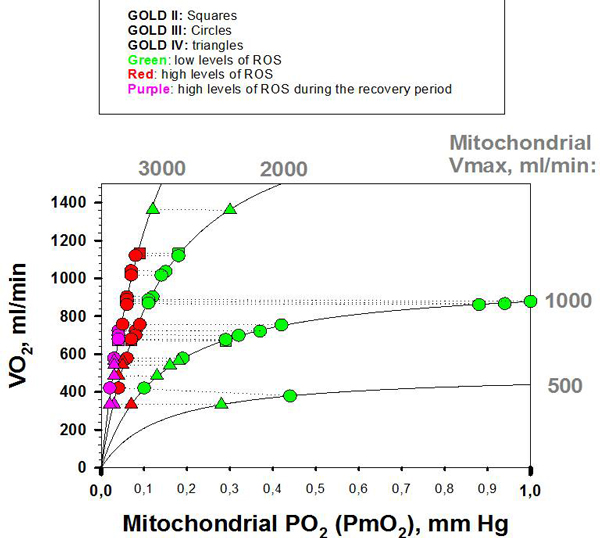
**Relationships between measured maximum O_2 _transport (VO_2_), y-axis; and, estimated cellular oxygenation (PmO_2_), x-axis, in COPD patients**. The different symbols correspond to classical GOLD stages: squares, GOLD II; circles GOLD III; and, triangles, GOLD IV (measured VO_2 _obtained from [32]). The symbols connected with discontinuous lines correspond to the same patient (same VO_2_) with estimated PmO_2 _values corresponding to different mitochondrial oxidative capacities (Vmax values and VO_2_/Vmax ratios). For a given patient, the lower the VO_2_/Vmax ratio, the lower was the estimated PmO_2_. The colors correspond to the mitochondrial ROS generation: green, mitochondrial ROS levels similar to those seen in healthy subjects; red, abnormally high mitochondrial ROS levels; and, violet, high ROS levels that persist after exercise withdrawal. The lower the PmO_2_, the higher were mitochondrial ROS levels.

**Skeletal muscle dysfunction in COPD patients**. Figure 3 displays the relationships between estimated skeletal muscle PO_2 _(PmO_2_) and mitochondrial ROS production for different levels of maximum O_2 _transport and mitochondrial utilization capacity in a group of COPD patients with mild to severe disease [32], as explained in the figure legend. The central messages of this analysis were: *i) *PmO_2 _at maximal exercise is determined by the ratio between O_2 _transport to mitochondrial and O_2 _utilization capacity (VO_2_max/Vmax ratio), such that the lower the maximum O_2 _transport potential for a given mitochondrial capacity, the lower PmO_2_; *ii) *tissue oxygenation levels were not related with GOLD stages; and, *iii) *low PmO_2 _values associated with abnormally high mitochondrial ROS production at peak exercise were predicted to occur in these patients. The analysis of the relative impact of the determinants of skeletal muscle oxygenation using the integrated model of O_2 _pathway and mitochondrial ROS generation also indicated: (1) how functional heterogeneities of skeletal muscle VO_2_max/Vmax ratios may generate both very low and very high PmO_2 _in the skeleletal muscle of these patients; and (2) the high impact of lung heterogeneity decreasing overall O_2 _transport, as compared with the rather moderate role of skeletal muscle heterogeneity on mean PmO_2_.

**Figure 4:**
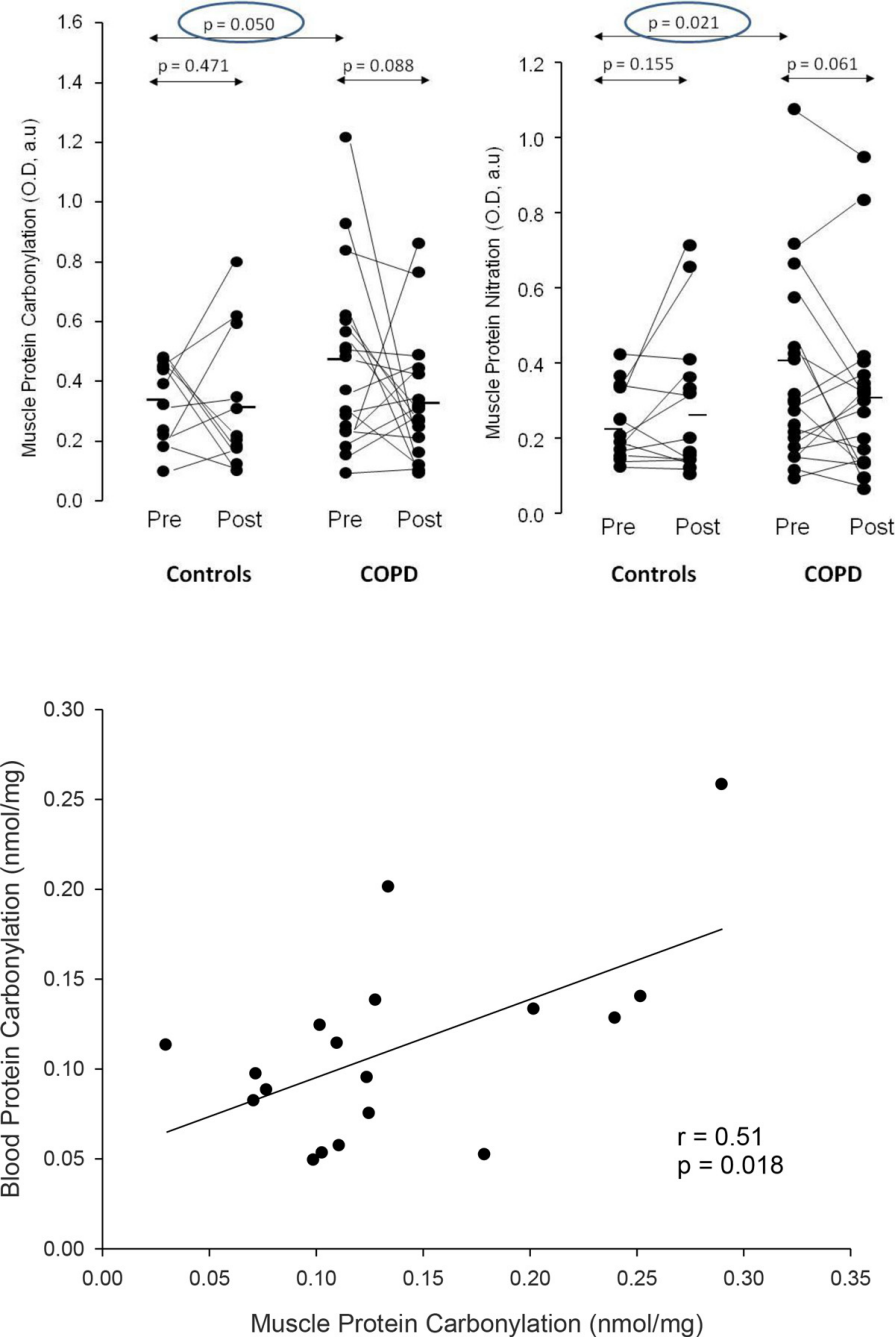
**Oxidative stress in COPD**. **Upper panel**: Muscle oxidative stress. Individual and mean group effects of an 8-week endurance training program on protein carbonylation (left) and protein nitration (right) in the vastus lateralis of healthy subjects (controls) and patients with COPD. At baseline (rest, pre-training measurements), COPD patients showed higher nitroso-redox disequilibrium than healthy subjects. A trend toward a decrease in oxidative stress was observed after training in COPD patients [28]. **Bottom panel**: Association between muscle and blood. COPD patients at baseline (rest, pre-training) showed an association of protein carbonylation levels between skeletal muscle and blood [28] (*reproduced from*[28]*with permission*).

**Figure 5:**
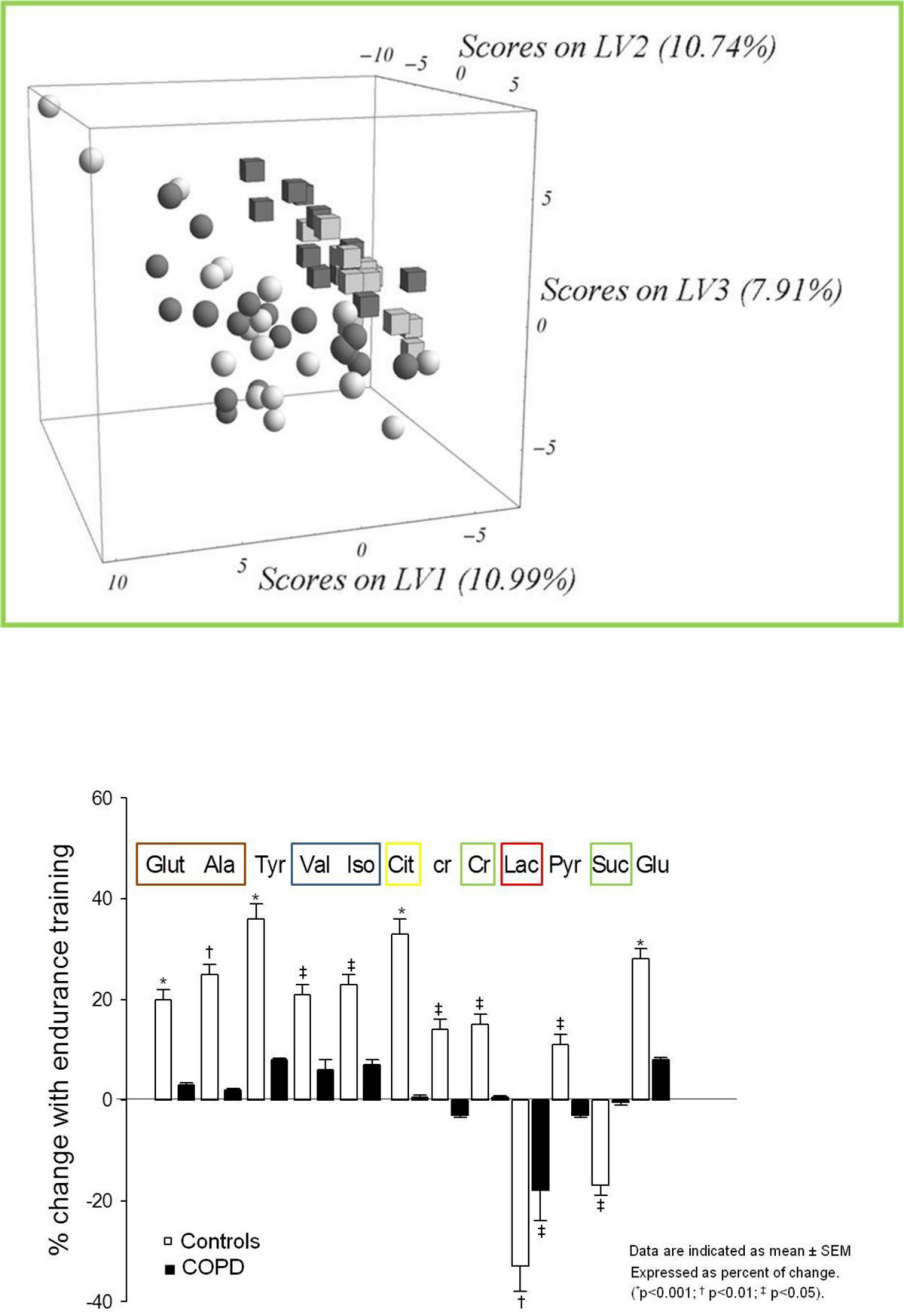
**Metabolic analysis**. **Upper panel**: Resting individual metabolic profiles in COPD patients (spheres) and in healthy sedentary subjects (cubes), including pre (black symbols) - and post -training data (grey symbols). The results are expressed by the three Latent Variables (LV1, 2 and 3) of the partial-least square discriminant analysis (PLS-DA). The percentages indicate the magnitude of the differences between the two groups of subjects for each dimension (p<0.05). **Bottom panel**: Endurance training responses of individual metabolites. Mean training-induced responses of individual metabolites. Data expressed as percent of change are indicated as mean ± SEM. (*p<0.001; ^† ^p<0.01; ^‡ ^p<0.05) *(reproduced from *[27]*with permission)*

The Biobridge dataset [27,28,33] clearly indicated that COPD patients at rest, before training, showed nitroso-redox disequilibrium both in blood and skeletal muscle compared to healthy controls (Figure 4, upper panel). Moreover, a significant association of protein carbonylation levels between blood and skeletal muscle was observed in the patients (Figure 4, bottom panel) [28] in whom low-grade inflammation in peripheral blood, but not in skeletal muscle, was observed. The plasma metabolomic analysis in COPD was fully consistent with abnormal skeletal muscle abnormalities reported in these patients [27], namely: decreased oxidative capacity leading to abnormal ROS generation [28,44,45], up-regulation of glycolysis [46] and altered aminoacid metabolism [27,46] (Figure 5). The transcriptomic analysis showed lower and abnormal skeletal muscle gene expression at baseline in COPD patients compared to healthy subjects with clear differences between COPD patients with preserved skeletal muscle mass and those showing muscle wasting [33].

**Figure 6:**
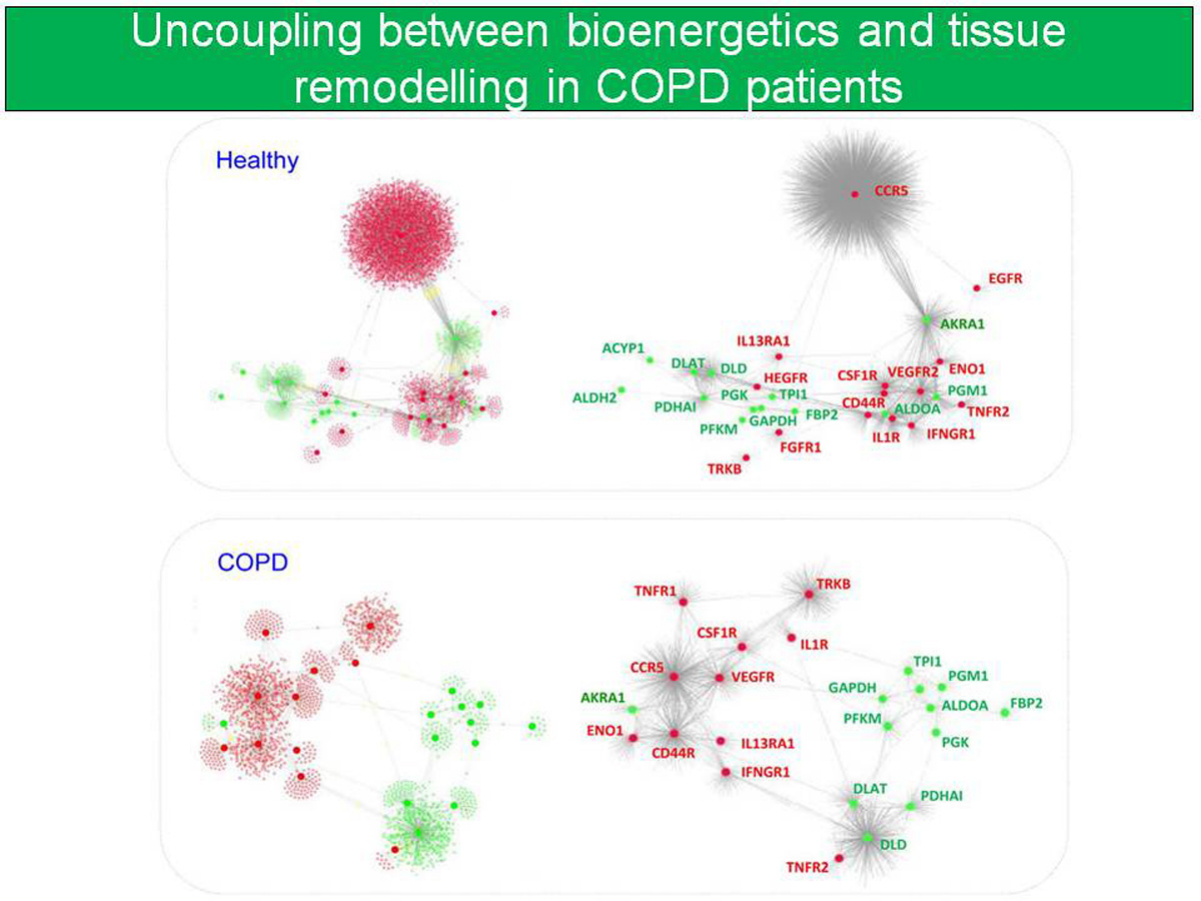
**Interaction networks using skeletal muscle expression profiling, plasma cytokines and physiological measurements**. Uncoupling between bioenergetics, inflammation and skeletal muscle remodeling was observed in COPD patients as compared to healthy subjects [33]

Most importantly, the network medicine approach assessing associations among three major pathways, namely bioenergetics, inflammation and skeletal muscle remodeling showed clear differences between the probabilistic models obtained in healthy subjects and those seen in COPD patients indicating failure to coordinately activate these pivotal metabolic pathways in the patients (Figure 6). Moreover, a sub-analysis carried out with few muscle samples identified a potential role of epigenetic changes contributing to the phenomenon of abnormal regulation of key metabolic pathways seen in COPD patients.

The study [27,28] demonstrated that high intensity 8-w endurance training significantly enhanced aerobic capacity both in healthy controls and in COPD patients without harmful effects on nitroso-redox equilibrium in severe COPD patients, but it confirmed abnormal training-induced skeletal muscle adaptations of the redox system in COPD indicated by a poor post-training increase of total gluthatione to oxidized gluthatione ratio in skeletal muscle seen in healthy subjects. On-going network analyses using the Biobridge dataset [27,28,33] further supports a pivotal role of nitroso-redox disequilibrium on skeletal muscle dysfunction in COPD patients.

**Co-morbidity clustering **- The large data driven analysis of co-morbidities indicated that COPD patients showed a higher risk for co-morbidity clustering than non-COPD patients of the same age. Not surprisingly, the likelihood for co-morbidity occurrence in COPD patients significantly increased with age for most conditions. Moreover, it was shown that specific cytokines and variables associated to the redox system [28,46] presented significant relationships with co-morbidity clustering in COPD patients. The network medicine analysis of the targeted co-morbidity clustering including CVD and T2D-MS is currently underway.

**Dissociation between airway remodeling and pulmonary emphysema **- The modeling of spatial pulmonary heterogeneities was used to explore the characteristics of patients from the PAC-COPD study [39] in whom moderate to severe emphysema score, assessed by high resolution CT scan, was not accompanied by significant central airway remodeling. Consequently, these COPD patients showed mild FEV_1 _impairment. Unfortunately, the maturity of the modeling developments did not allow completion of the analysis as initially planned, see [40] for further details.

### Tackling COPD heterogeneities to enhance health outcomes

As part of the strategies for transferring novel biomedical knowledge into the clinical arena, we developed three families of CDSS (Table 2) that were embedded into the clinical processes at primary care level using an Integrated Care Shared Knowledge Platform [41] as technological support to facilitate the management of chronic patients. The CDSS' rules combined existing and novel knowledge generated during the project's life-span, as explained in detail in [42]. The CDSS areas addressed in the project are briefly described below:

**Early diagnosis of COPD **- A COPD case-finding program for citizens with high risk for developing COPD was deployed at local level [41]. The program encompasses different aspects: *i) *remote support to automatic assessment of quality of forced spirometry both in pharmacy offices and primary care [47]; *ii) *support to coordination between informal care (pharmacy offices) and formal care (primary care and specialists); and, *iii) *enhanced 2011 GOLD-based COPD assessment with the use of recommended reference equations [48].

**Enhanced stratification of COPD patients**. A suite of CDSS supporting proposals for patient stratification has been built-up. The CDSS facilitates comparisons with available predictive indices for COPD patients. That is, classical GOLD staging (groups 1 to 4 based on FEV_1 _% predicted) and 2011 GOLD update (groups A to D), taking into account symptoms score and frequency of severe exacerbations, can be automatically constructed and displayed in the primary care clinical workstation. Co-morbidities expressed as number&type and Charlson index are also considered together with novel proposals for decision algorithms based on the Synergy-COPD findings, as detailed in [42].

**Community-based integrated care management of COPD patients**. Deployment experiences of integrated care services [41] developed in parallel with Synergy-COPD have demonstrated positive health outcomes together with cost-containment through the transfer of healthcare complexity from specialized care to the community fostering an active and participatory role of both citizens at risk, patients and carers. In this scenario, the use of CDSS to support health professionals for chronic care management appears as an effective approach to transfer novel biomedical knowledge into healthcare. Such an approach was assessed in the validation work package of the project. Moreover, the parallel deployment experiences [41] carried out during the life time of the project identified the high potential of the Personal Health Folder (PHF) [49] for transferring different types of non-medical patient information, namely: life-styles, social frailty, adherence profile, etc... into formal healthcare, as detailed in [50].

### Lessons learnt to foster 4P Medicine of chronic conditions

Synergy-COPD proposes a comprehensive strategy to foster the interplay between system research and predictive medicine for chronic conditions. This purpose led to the inception of the Digital Health Framework (DHF) extensively presented in [50] of the current Supplement, as the Synergy-COPD's proposal to favor iterations between informal care, formal care and biomedical research.

## Discussion

### Biomedical contributions to COPD heterogeneity

The overall biological findings generated by the different studies associated with the project support the concept that the pulmonary events of the disease are not the only driver, and sometimes neither the main one, of the disturbances seen in these patients and do not explain by themselves COPD heterogeneity. Instead, a constellation of concurrent factors (systemic effects of tobacco, lack of physical activity, unhealthy diet, patient susceptibility to oxidative stress, cellular hypoxia, etc...) may lead to systemic nitroso-redox disequilibrium and low-grade blood inflammation seen in COPD patients with skeletal muscle dysfunction/wasting, as well as in those with co-morbidities.

There is evidence supporting that the combination of the subject's genetic susceptibility, changes in functional genomics, partly regulated by epigenetic events, together with changes in post-translational regulatory phenomena at several levels may modulate the impact of the factors triggering the disease and ultimately may determine the clinical manifestations, as well as the rate of progression of the different COPD components: pulmonary, systemic effects and co-morbidity clustering. Moreover, it can be speculated that the tight multilevel regulation of aerobic capacity in man [26], may constitute a relevant factor explaining the interdependence among the three drivers of COPD heterogeneity. As a simplified example, down-regulation of the skeletal muscle tricarboxilic acid cycle (TCA) metabolism together with up-regulation of glycolysis have been extensively reported in COPD patients with skeletal muscle dysfunction [10] as a leading cause of early lactate release during moderate exercise. The phenomenon overloads the ventilatory function facilitating air trapping and shortness of breath often contributing to sedentarism which, in turn, may constitute one of the contributing factors to insulin resistance, and T2DM, often seen in these patients. Obviously, the progression of pulmonary disease severity contributes to the vicious circle because it worsens the underlying mechanisms involved in the systemic effects of COPD and the co-morbidity clustering.

The unifying hypothesis proposed by Synergy-COPD is supported by all the findings observed in the different studies carried out in the project. Moreover, it is consistent with different reports in the literature [26,44] indicating that preserved aerobic function seems to be associated with nitroso-redox equilibrium in the cardiovascular system and, consequently, with O_2 _flow to O_2 _uptake matching in peripheral tissues. All in all, the novel view of COPD heterogeneity proposed by the Synergy-COPD project has profound implications in terms of patient health risk assessment and stratification, but most importantly, it may deeply modulate both management and therapeutic approaches of these patients.

The 2014 report [6] analyzing the clinical implications of the ECLIPSE results strengthen the relevance of COPD heterogeneity on the clinical management of these patients. However, the highly valuable ECLIPSE's achievements favoring a new clinical vision of COPD essentially have a descriptive nature with well recognized limitations in terms of generating evidence on novel underlying disease mechanisms.

In summary, there are three relevant lessons learnt both from the project itself and from recent contributions like ECLIPSE:

• The characteristics of COPD heterogeneity clearly generate a mandate for the design of strategies aiming at individual health risk assessment and patient-oriented management stratification aiming at setting cost-effective preventive interventions to modulate disease progress.

• The unifying hypothesis for COPD heterogeneity explored in Synergy-COPD contains the core elements for the design of a coherent patient stratification strategy, as proposed in the current manuscript.

• The still on-going network analyses in Synergy-COPD may generate "in silico" hypothesis on the specifics of underlying mechanisms of COPD heterogeneity to be implemented in further refined versions of the proposed CDSS.

### Modeling tools and strategies

The project has generated important modeling outcomes and it has identified relevant challenges to be faced beyond the project lifetime. Moreover, Synergy-COPD has also shaped specific approaches to face those challenges, as extensively discussed in [31]. We acknowledge, however, limited achievements in terms of identification of combined biomarkers with predictive power, as well as in the development of subject-specific predictive modeling to feed the CDSS for the reasons discussed in other areas of the Supplement. We also recognized limited achievements in the targeted objectives relative to pulmonary events. There is no doubt that the positive interactions with AIRPROM will contribute to a maturity of the modeling of pulmonary spatial heterogeneities described in [40] allowing to further undertake this specific challenge.

### Logistics for 4P medicine

The accepted limitations in terms of subject-specific predictive modeling did not preclude other relevant technological and organizational outcomes such as the developments of novel CDSS [42] and the formulation of the Digital Health Framework (DHF) [50]. We believe that the deployment of these tools within an integrated care scenario paves the way toward predictive, preventive, participatory and personalized (4P) medicine for these patients preventing fragmentation of care. It is important to note that the entire DHF [50] still requires a proof-of-concept validation before considering specific strategies for its deployment.

The transition toward a novel biomedical research scenario fostering 4P medicine has two major biomedical research goals, namely: *i) *to speed-up the transfer of biomedical knowledge, including novel therapies, into healthcare; and. *ii) *to generate operational feedback from healthcare and informal care into biomedical research. The last step shall generate two main added values. Firstly, biological knowledge will be enriched with information on different dimensions of the patient (adherence profile, frailty, life styles, socio-economical and environmental factors, etc...) and, secondly, it will facilitate an iterative process that shall result in progressive refinement of subject-specific predictive modeling. In this regard, the interoperability among the PHF, the Integrated Care Shared Knowledge Platform and the novel biomedical research platform proposed in [50], within the concept of the DHF, constitutes a major achievement of the project toward the consolidation of innovative biomedical research scenario that overcomes current limitations due to fragmentation of the information.

## Conclusions

The systems approach to COPD heterogeneity explored in the Synergy-COPD project has generated a novel view of the phenomenon wherein systemic effects of the disease and co-morbidity clustering, may all have a relevant role in the COPD patients, independently from pulmonary events. The interdependence between pulmonary and extra-pulmonary events was formulated. Moreover, the chapter assessed specific strategies for implementation of the system approach into integrated care management for chronic patients. Finally, the impact of the novel vision into biomedical research was explored.

## Competing interests

The authors declare they have no competing interests.

## Declarations

The research described in this paper is partly supported by the Synergy-COPD European project (FP7-ICT-270086). Publication of this article has been funded by the Synergy-COPD European project (FP7-ICT-270086).

This article has been published as part of *Journal of Translational Medicine *Volume 12 Supplement 2, 2014: Systems medicine in chronic diseases: COPD as a use case. The full contents of the supplement are available online at http://www.translational-medicine.com/supplements/12/S2.
